# Extraction of ketamine and dexmedetomidine by extracorporeal life support circuits★

**DOI:** 10.1051/ject/2024016

**Published:** 2024-09-20

**Authors:** Andrew Chevalier, J. Porter Hunt, Aviva Whelan, Autumn McKnite, Kevin M. Watt, Danielle J. Green

**Affiliations:** 1 Division of Pediatric Critical Care, Department of Pediatrics, University of Utah Salt Lake City UT 84132 USA; 2 Division of Clinical Pharmacology, Department of Pediatrics, University of Utah Salt Lake City UT 84132 USA; 3 Department of Pharmacology and Toxicology, University of Utah Salt Lake City UT 84132 USA

**Keywords:** ECMO, Pharmacology, Dialysis, Sedation, ECLS

## Abstract

*Background*: Patients supported with extracorporeal life support (ECLS) circuits such as ECMO and CRRT often require high doses of sedatives and analgesics, including ketamine and dexmedetomidine. Concentrations of many medications are affected by ECLS circuits through adsorption to the circuit components, dialysis, as well as the large volume of blood used to prime the circuits. However, the impact of ECLS circuits on ketamine and dexmedetomidine pharmacokinetics has not been well described. This study determined ketamine and dexmedetomidine extraction by extracorporeal circuits in an *ex-vivo* system. *Methods:* Medication was administered at therapeutic concentration to blood-primed, closed-loop *ex-vivo* ECMO and CRRT circuits. Drug concentrations were measured in plasma, hemofiltrate, and control samples at multiple time points throughout the experiments. At each sample time point, the percentage of drug recovery was calculated. *Results*: Ketamine plasma concentration in the ECMO and CRRT circuits decreased rapidly, with 43.8% recovery (SD = 0.6%) from ECMO circuits after 8 h and 3.3% (SD = 1.8%) recovery from CRRT circuits after 6 h. Dexmedetomidine was also cleared from CRRT circuits, with 20.3% recovery (SD = 1.8%) after 6 h. Concentrations of both medications were very stable in the control experiments, with approximately 100% drug recovery of both ketamine and dexmedetomidine after 6 h. *Conclusion*: Ketamine and dexmedetomidine concentrations are significantly affected by ECLS circuits, indicating that dosing adjustments are needed for patients supported with ECMO and CRRT.

## Introduction

Extracorporeal life support (ECLS) circuits, including extracorporeal membrane oxygenation (ECMO) and continuous renal replacement therapy (CRRT), are potentially life-saving therapies for patients with refractory organ failure [1–3]. Almost universally, patients who are supported with ECLS require sedation to maintain patient safety and comfort [4] Drug exposure resulting from standard dosing of these medications may be significantly affected by ECLS circuits. ECLS circuits alter medication concentrations in the following ways: 1) adsorption of medication to circuit components; 2) clearance of medication via dialysis; and 3) increased volume of distribution from a circuit prime [5]. Medications that are highly protein-bound and lipophilic tend to be most affected by adsorption; hydrophilic, minimally protein-bound medications tend to be cleared by dialysis [5, 6]. As sedatives tend to be lipophilic, a high degree of interaction with ECLS circuits is expected. Understanding these interactions is critical to avoiding treatment failure.

Ketamine and dexmedetomidine are two sedating medications that are recommended by national guidelines for use in critically ill patients [7, 8]. Ketamine, an NMDA receptor antagonist, is a medication that has sedating, amnestic, and analgesic effects. In contrast to most sedatives, ketamine does not tend to lower blood pressure, making it especially useful for patients at risk of hemodynamic instability, such as those on ECLS. Additionally, ketamine confers the added benefit of bronchodilation, thus leading to its use in refractory status asthmaticus. Finally, ketamine has been demonstrated to have an opioid and benzodiazepine-sparing effect [9]. Unfortunately, if used in excess, ketamine may contribute to hypertension, tachycardia, excess pulmonary secretions, or worsened delirium. Dexmedetomidine, an agonist at central alpha-2 receptors, is an effective and safe sedative that has been noted in multiple studies to be associated with a reduced risk of delirium compared with opioids and benzodiazepines [8]. Excess dexmedetomidine may cause bradycardia and hypotension [10]. While dexmedetomidine is significantly extracted by ECMO circuits in *ex-vivo* experiments [11], data guiding the use of dexmedetomidine in CRRT or the use of ketamine in ECMO or CRRT is limited [11–13]. Because few studies exist to guide the dosing of ketamine and dexmedetomidine for patients with ECLS, a study on the interactions of ketamine and dexmedetomidine with ECLS circuits is needed.

In this study, we quantify the degree of interaction of dexmedetomidine and ketamine with ECLS circuits using *ex-vivo* ECMO and CRRT experiments.

## Materials and methods

Circuit-drug interactions were evaluated in closed-loop ECMO and CRRT circuits similar to previously published *ex-vivo* models [14–21]. Briefly, ECMO and CRRT circuits were constructed using common circuit components and a reservoir primed with a human blood and plasma mixture. Ketamine and dexmedetomidine were then injected into the circuits targeting a therapeutic concentration, and the percentage of the drug remaining was determined over time.

### ECMO circuit setup

ECMO experiments were performed using a Maquet centrifugal pump with bioline coating (Maquet, Rastatt, Germany), Maquet Quadrox-iD oxygenator with bioline coating (Maquet, Rastatt, Germany), and Medtronic custom perfusion system tubing and reservoir (Medtronic, Minneapolis, Minnesota) (Table 1). The reservoir was primed with 2 units of human red blood cells (adenine saline added leukocytes reduced), 1 unit of thawed human plasma frozen within 24 h after phlebotomy, 12.5 grams of 25% albumin, 325 mg of calcium gluconate, 14 mEq of sodium bicarbonate, 1 gram of tromethamine (THAM), and 250 units of heparin sulfate. Additional THAM or carbon dioxide was added to the circuit prior to ketamine administration and as-needed hourly thereafter to maintain physiologic pH 7.20-7.45 as measured on an i-STAT Analyzer (300-G, Flextronics Manufacturing, Singapore) using EG6+ cartridges (03P77-25, Abbott Point of Care, Abbott Park, IL). The temperature of the circuit was maintained at 37 degrees C using an ECMO Water Heater (Cincinnati Sub-Zero, Cincinnati, OH) connected to the Quadrox-iD integrated heat exchanger. Flow through the ECMO circuit was maintained at 1.0 L/min measured with an HT110 bypass flowmeter with an H8XL flow sensor (Transonic, Davis, CA). The flow was maintained at 1.0 L/min as this is approximately the flow that would be used for a 10 kg child flowing at a typical flow of 100 ml/kg/min.

**Table 1:** Circuit components.

Circuit type	Component	Manufacturer	Model	Material
ECMO	Reservoir	Medtronic	Medtronic Custom Perfusion Set	Medtronic cortiva^a^
	Tubing	Medtronic	Medtronic Custom Perfusion Set	Medtronic cortiva^a^
	Pump	Maquet	Rotaflow centrifugal pump	Polymethylpentene hollow fibres with bioline^b^ coating
	Oxygenator	Maquet	Quadrox-iD adult oxygenator	Polymethylpentene hollow fibres with bioline^b^ coating

CRRT	Reservoir	Baxter	EXACTAMIX EVA, 500 ml	Ethylene vinyl acetate
	System	Baxter	Prismax with TherMax heater	N/A
	Hemofilter	Baxter	HF1000	Polyarylethersulfone hollow fibers, plasticized polyvinyl chloride tubing
	Warmer bag	Baxter	Thermax blood warmer	Polyurethane

a Cortiva coating: heparin covalently bonded to polymer.

b Bioline coating: covalently bonded recombinant human albumin and heparin.

### CRRT circuit setup

CRRT experiments were performed using a Baxter Prismax system (Baxter Healthcare, Deerfield, IL) with a THERMAX blood warmer (Baxter Healthcare, Deerfield, IL) and HF1000 filter (Baxter Healthcare, Deerfield, IL) (Table 1). A 500 ml Baxter EXACTAMIX (Baxter Healthcare, Deerfield, IL) bag was used as a reservoir for the circuit and was continuously stirred by an orbital shaker at 80 rpm. The reservoir was primed with 300 ml of human red blood cells (adenine saline added leukocytes reduced), 125 ml of thawed human plasma within frozen 24 h after phlebotomy, 12.5 grams of 25% albumin, 180 mg of calcium gluconate, 7 mEq of sodium bicarbonate, 1.4 g of THAM, and 350 units of heparin sulfate, and this mixture was then connected to the circuit and pumped through the circuit. Additional THAM was added prior to ketamine or dexmedetomidine administration and hourly thereafter as needed to maintain physiologic pH 7.2-7.45 as measured on an i-STAT Analyzer using EG6+ cartridges. Temperature was maintained at 37.0 degrees C by the THERMAX blood warmer. Circuits ran in continuous venovenous hemodiafiltration (CVVHDF) circuit configuration with both dialysate and replacement fluids as this is the most common configuration used at our institution. Dialysis circuits used a blood flow rate of 80 ml/min, pre-blood pump (PBP) replacement fluid rate of 300 ml/h, post-filter replacement fluid rate of 100 ml/h, and dialysate fluid rate of 400 ml/h, with a net ultrafiltration rate of 0 ml/h. PrismaSATE 4/2.5 dialysis solution (Baxter Healthcare, Deerfield, IL) was used for dialysis, pre-blood pump, and replacement fluids. Due to premature circuit failure in two circuits, a total of 5 CRRT circuits were conducted for ketamine. Dexmedetomidine CRRT circuits were run in triplicate.

### Control setup

For the ketamine experiments, 50 ml of the ECMO prime solution was placed in a 50 ml polypropylene centrifuge tube (CELLTREAT, Pepperell, MA) to serve as a control solution to measure medication stability over the time course of the experiments. The solution was incubated in a water bath at 37 degrees C. For the dexmedetomidine experiments, data from ECMO circuit *ex-vivo* experiments previously published by our group served as the control. In these experiments, dexmedetomidine concentrations were demonstrated to be stable over time, with 102% recovery after 24 h [15].

### Medication administration and sample collection

Ketamine and dexmedetomidine were obtained from the University of Utah hospital pharmacy. Medication was dosed to the circuit experiments and the control experiments targeting therapeutic concentrations for ketamine (200 ng/ml) [22] and dexmedetomidine (1.6 ng/ml) [23].

**Figure 1:**
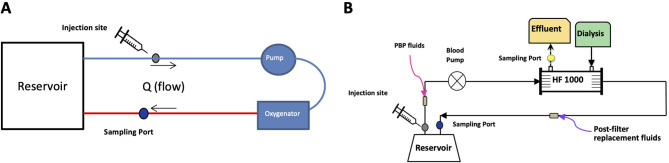
Schematic of ECMO and CRRT *ex-vivo* circuit configurations. (A) ECMO circuit experiment demonstrating layout of reservoir, tubing, pump and oxygenators and locations of injection and sampling sites. (B) CRRT circuit experiment demonstrating CVVHDF configuration as well as location of injection and sampling sites. PBP = pre-blood pump.

For the ECMO circuits, medication was administered via a three-way stopcock in the venous limb (Figure 1A). Blood samples were collected from the arterial limb of the circuit 1, 5, 15, and 30 min, and 1, 2, 3, 4, 5, 6, and 8 h after medication administration. Experiments were performed in triplicate.

For the CRRT circuits, medication was administered via a three-way stopcock on the access line downstream from the reservoir. Blood samples were drawn from a stopcock via the return line just upstream from the reservoir and hemofiltrate samples were drawn from a stopcock just upstream from the effluent bag (Figure 1B). Paired blood and hemofiltrate samples were collected at 1, 5, 15, and 30 min, and 1, 2, 3, 4, 5, and 6 h after medication administration. Due to multiple premature circuit failures for the ketamine experiments, three runs were performed out to 6 h, one circuit ran for 5 h, and one circuit ran for 2 h. Dexmedetomidine experiments were performed in triplicate.

Medication was directly added to the control experiments followed by 5 min of mixing on a rotary mixer prior to sample collection. Samples were subsequently collected at 1, 5, 15, and 30 min, and 1, 2, 3, 4, 5, 6, and 8 h, with gentle inversion prior to sample collection to ensure adequate mixing.

All blood samples were centrifuged at 3000 g at 4 degrees C for 10 min. The plasma was manually pipetted off and immediately frozen in cryovials (Fisher Scientific, Pittsburgh, PA) and stored at −80 degrees C until analysis. Hemofiltrate samples were pipetted directly into cryovials (Fisher Scientific, Pittsburgh, PA) and stored at −80 degrees C until analysis.

### Sample analysis

Plasma and hemofiltrate samples for the ketamine and dexmedetomidine experiments were analyzed at OpAns Laboratory (Durham, NC) using high-performance liquid chromatography combined with tandem mass spectrometry (HLPC – MS/MS). The range of quantitation for the ketamine and dexmedetomidine assays was 1 ng/ml-10,000 ng/ml and 25 pg/ml-10,000 pg/ml respectively.

### Percent recovery calculation

To correct for variations in initial drug concentration due to small differences in circuit volume, drug percent recovery for each sample collection was calculated using the following equation:

$$\mathrm{Recovery}\;\left(\%\right)=\;\frac{C_{t}}{C_{\mathrm{ref}}}\times 100\%$$

where *C*_t_ was the concentration at time t, and C_ref_ was the reference concentration of the drug in the circuit. For both the ECMO experiments and the CRRT experiments, medication was not detected in the plasma samples at 1 minute as circuit mixing was not immediate. As a result, the concentration at 5 min was used as the reference concentration for both the circuit experiments and the control experiments.

### Hemofiltrate calculations

The saturation coefficient for CRRT was calculated using the following equation:

$$S_{a}=\frac{C_{\mathrm{eff}}}{C_{p}}$$

where *S*_a_ is the saturation coefficient, *C*_eff_ is the effluent concentration, and *C*_p_ is the plasma concentration. The saturation coefficient was calculated at each timepoint from 15 min onwards as this allowed for time for medication equilibration throughout the hemofiltrate.

Dialysis clearance was determined by the following equation:

$$\mathrm{Cl}=Q_{\mathrm{eff}}\times S_{a}$$

where Cl is the clearance via dialysis, *Q*_eff_ is the total effluent flow, and *S*_a_ is the saturation coefficient.

### Statistics

Statistical analysis was performed using the statistical software R, with the addition of the Tidyverse, Ggpubr, and Rstatix packages [24–26]. A two-sample t-test with an alpha level of 0.05 was used to compare the plasma percent recovery between the circuits and the control samples after the experiment.

## Results

### Ketamine ECMO circuits

**Figure 2:**
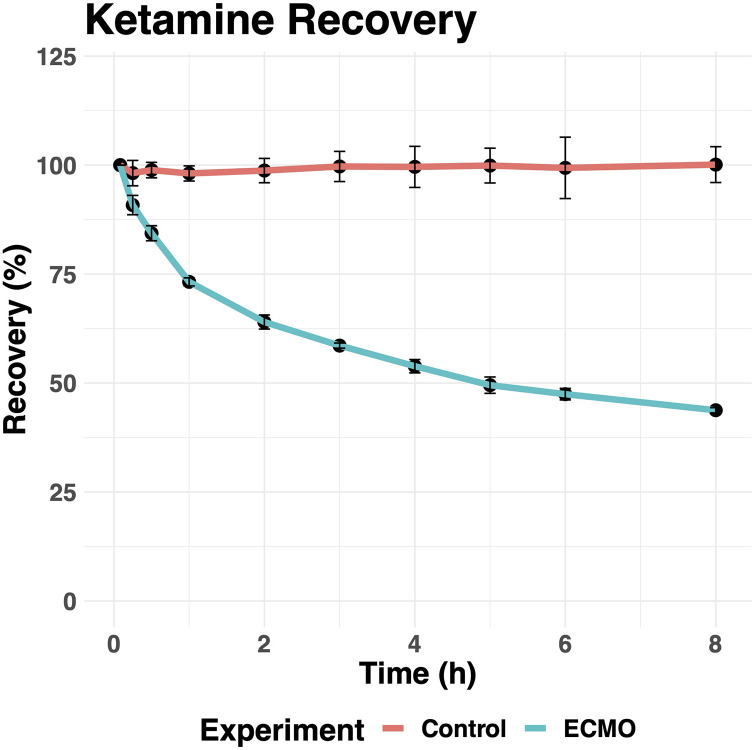
The percent recovery of ketamine from control (red) and *ex-vivo* ECMO circuits (blue) over time. Error bars represent one standard deviation *n* = 3 control and *n* = 3 experimental circuits.

Ketamine concentrations decreased rapidly in the ECMO circuits (Figure 2). The mean recovery of ketamine from ECMO circuits was 43.8% (*n* = 3, SD = 0.6%) after 8 h. In contrast, ketamine levels remained relatively constant over time in the control sample with 100% (*n* = 3, SD = 4%) recovery after 8 h, a difference that reached statistical significance (*p* < 0.001).

### Ketamine and dexmedetomidine CRRT circuits

**Figure 3:**
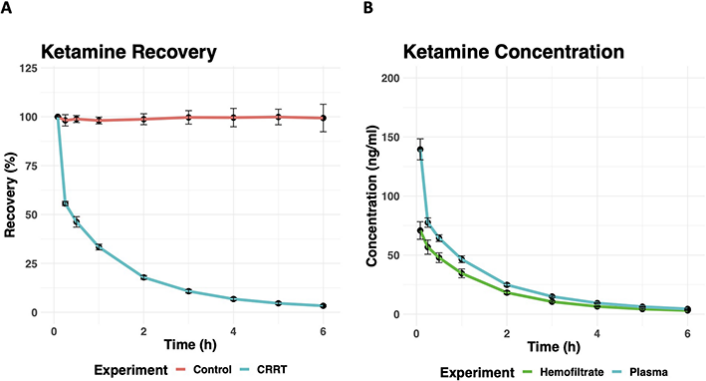
(A) The percent recovery of ketamine from control (red) and *ex-vivo* CRRT circuits (blue) over time. Error bars represent one standard deviation for *n* = 3 control and *n* = 5 experimental circuits. (B) The concentration of ketamine in plasma (blue) and hemofiltrate (green) of *ex-vivo* CRRT circuits over time. Error bars represent one standard deviation for *n* = 3 control and *n* = 5 experimental circuits.

Similar to the ECMO circuits, ketamine was rapidly cleared from CRRT circuits (Figure 3A) with a mean recovery of 3.3% (*n* = 3, SD = 0.5%) after 6 h. Percent recovery of ketamine was significantly different from the control samples (*p* < 0.001). Ketamine concentrations in hemofiltrate were similar to those in plasma (Figure 3B) with a mean saturation coefficient of 0.72 (SD = 0.06), corresponding to a transmembrane clearance of 9.6 ml/min at an effluent rate of 800 ml/h in our experiment.

**Figure 4:**
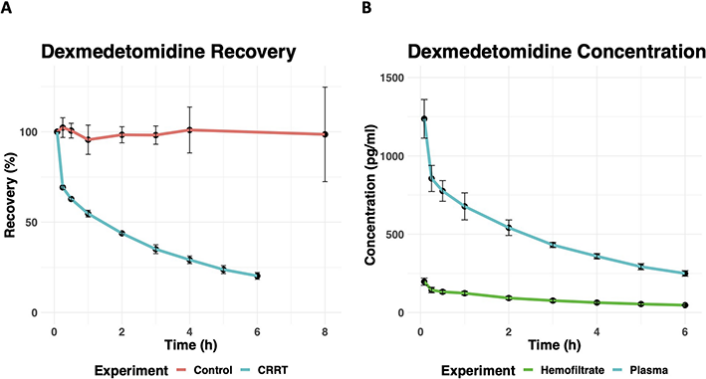
(A) The percent recovery of dexmedetomidine from control (red) and *ex-vivo* CRRT circuits (blue) over time. Error bars represent one standard deviation for *n* = 9 control and *n* = 3 experimental circuits. (B) The concentration of dexmedetomidine in plasma (blue) and hemofiltrate (green) of *ex-vivo* CRRT circuits over time. Error bars represent one standard deviation for *n* = 9 control and *n* = 3 experimental circuits.

Compared to ketamine, dexmedetomidine was more slowly cleared from CRRT circuits (Figure 4A) with a mean recovery of 20.3% (*n* = 3, SD = 1.8%) after 6 h. In contrast, the previously reported data for dexmedetomidine control experiments revealed a mean recovery of 99% (*n* = 9, SD = 26%) at 4 h [15]. The average saturation coefficient was 0.18 (SD = 0.01), corresponding to a transmembrane clearance of 2.4 ml/min at the effluent flow rate used in our experiments (Figure 4B).

## Discussion

These experiments demonstrated significant interactions between ECLS circuits and two common sedatives, ketamine and dexmedetomidine.

Ketamine recovery from ECMO circuits was significantly lower than control samples. ECMO circuit extraction was likely related to the adsorption of ketamine to circuit components, including the oxygenator, pump, and tubing. This degree of circuit interaction confirms our hypothesis that ketamine’s relative lipid solubility (logP = 3.12) [27] leads to significant circuit adsorption and suggests the need for dosing adjustments of ketamine for patients on ECMO. These findings differ somewhat from prior literature [11, 12, 28–30]. One case report found a higher volume of distribution and clearance for ketamine in an adult patient supported by ECMO compared to healthy patients [29]. However, these pharmacokinetic parameters were similar to those reported in critically ill patients not on ECMO [29]. A second case report of an adult patient on ECMO observed target sedation levels after standard doses of a ketamine infusion, although goal sedation occurred at lower ketamine concentrations than standard [30]. Importantly, these reports cannot distinguish the degree to which differences in ketamine exposure and/or response result from the ECMO circuit itself or the underlying critical illness of the patient. While these reports could suggest that ketamine dosing adjustments are not required, additional research is required to reveal the extent to which ketamine dosing should be adjusted in patients on ECMO.

Ketamine recovery from CRRT circuits declined rapidly. Some of the ketamine extraction was presumably related to clearance across the dialysis membrane, with a relatively high saturation coefficient of 0.72. This degree of ketamine filtration is the result of ketamine’s moderate degree of protein binding (54%) [27], with almost half of the medication unbound and available for filtration. Prior studies of ketamine pharmacokinetics in CRRT are limited to a single identified case series [31]. This study revealed similar *in-vivo* transmembrane clearance and saturation coefficients across dialysis filters as noted in our study but did not report on the degree of circuit absorption. The rapid alterations in ketamine levels observed in our experiments would suggest that alterations in dosing are needed.

Dexmedetomidine concentrations were significantly altered by the CRRT circuit, albeit at a slower rate than ketamine. Clearance across the dialysis membrane was minimal, with a saturation coefficient of 0.18. Dexmedetomidine is as lipid-soluble as ketamine (logP = 2.8) [32], but with significantly higher protein binding (94% vs. ~50%) [32]. Therefore, the fraction of unbound dexmedetomidine is expected to be much lower than for ketamine, contributing to the lower clearance across the membrane. With the low degree of membrane clearance, the majority of dexmedetomidine extraction by the CRRT circuit is theoretically due to adsorption to circuit components. We did not identify prior studies of dexmedetomidine pharmacokinetics during CRRT [13]. However, our results were consistent with previous *ex-vivo* studies of dexmedetomidine pharmacokinetics in ECMO, which revealed a high degree of adsorption to circuit components [11, 15, 33–35]. These results suggest that, despite lower transmembrane clearance, dexmedetomidine dosing adjustments should be considered in patients supported by CRRT.

These experiments have several limitations. First, these experiments utilized only a single model for each circuit component to mimic the components most frequently used at our institution; as a result, these experiments are not necessarily reflective of interactions between these medications and alternative oxygenators or dialysis filters. Additionally, all CRRT circuits used a CVVHDF modality; thus, results are not necessarily reflective of alternative modalities such as CVVHD or CVVHF. However, prior *ex-vivo* experiments for other drugs have not demonstrated significant differences in medication clearance across dialysis modalities, with the total effluent dose being the impactful determinant of clearance [17, 21]. Finally, experiments were conducted using a single, identical dose. As such, these experiments did not determine whether circuit adsorption can be saturated. However, ECMO oxygenators and CRRT filters have very large surface areas with a high availability of binding sites; thus, saturation of circuit binding sites would be unlikely without exceedingly large doses of medications [16].

Our findings indicate that patients supported with ECLS likely require higher than standard doses of ketamine and dexmedetomidine to achieve therapeutic effects. While these data cannot be directly extrapolated to quantitative dose adjustments, the results of these experiments can be used to calculate pharmacokinetic parameters for ECMO and CRRT, allowing for the generation of ECLS compartments in physiologically-based pharmacokinetic models. These models can then be used to generate dosing recommendations, which can in turn be validated through clinical pharmacokinetic studies [36].

## Data Availability

All data are available upon request to the corresponding author.
